# Molecular Mechanisms of Atopic Dermatitis Pathogenesis

**DOI:** 10.3390/ijms22084130

**Published:** 2021-04-16

**Authors:** Jowita Sroka-Tomaszewska, Magdalena Trzeciak

**Affiliations:** Department of Dermatology, Venereology and Allergology, Medical University of Gdansk, 80-214 Gdańsk, Poland; jsroka@uck.gda.pl

**Keywords:** atopic dermatitis, allergic diseases, pathogenesis, genetic disorders, epidermal barriers defects, immunological disturbances, microbiome, targeted therapies

## Abstract

Atopic dermatitis is a chronic, non-infectious inflammatory dermatosis. Acharacteristic feature is persistent itching of the skin. The chronic, relapsing course of the disease, economic burden, and the whole family’s involvement in the treatment process immensely reduce the quality of life of patients and their families. The disease emerges as a social problem by increasing indirect costs, such as visiting a doctor, absenteeism from work and school, and avoiding social interactions. Thepathophysiology of atopic dermatitis is complex and multifactorial. It includes genetic disorders, a defect in the epidermal barrier, an altered immune response, anddisruption of the skin’s microbial balance. The numerous complex changes at thegenetic level and innate and adaptive immunity provide the basis for characterizing the various phenotypes and endotypes of atopic dermatitis. Emerging therapies rely on the action of specific molecules involved in the disease’s pathogenesis. It may be the starting point for the individualization of atopic dermatitis treatment. This paper will try to present some molecular mechanisms of atopic dermatitis and their clinical implications.

## 1. Introduction

Atopic dermatitis (AD) is a recurrent, chronic, non-infectious inflammatory dermatosis characterized by persistent itching of the skin. It occurs mainly in the pediatric population, with a frequency of up to ~20%in this group of patients [1,2]. The incidence has been increasing steadily for several decades, not only in countries with a higher degree ofurbanization and economy but also in developing countries [3]. Such epidemiological transformation causes AD is one of the most common skin diseases in childhood. The disease develops in 50–60% of cases within the first year of life, and 90% of patients are up to five. Adults also suffer from AD, mostly from childhood, and there are also new adulthood cases [4]. Theclinical picture includes eczema-like eruptions, such as erythema, papules, exudative lesions of a specific location, depending on the patient’s age (infant, childhood andadulthood) andvarious skin degrees of dryness [5]. Due to the disease’s long-term course, chronic or recurrent inflammation and scratching come to skin thickening andlichenification. An inherent symptom of AD is persistent itching of the skin, which interferes with daily activity and causes insomnia and sleep disorder.This may significantly decrease the quality of life [6,7]. Itching of the skin is one of the primary diagnostic criteria of Hanifin and Rajka. Those are the most recognized and most frequently used medical practice standards to diagnose AD for epidemiological purposes, research and clinical trials [8]. Patients diagnosed with atopic eczema show an increased incidence of other allergic diseases. [9,10]. Atopic dermatitis develops first, then other manifestations of allergies such as food allergy, asthma and allergic rhinitis may appear. This disease sequence was called the allergic march (also called the atopic triad) [11]. Recently, the relationships andcoexistence with other pathologies such as internal diseases, including hypertension, diabetes or heart disease, as well as autoimmune diseases and mental disorders, have also been studied [9,10,12]. This research suggests a positive correlation between the degree of AD’s severity and the prevalence of these diseases [9,10]. The chronic, relapsing course of the disease, economic burden, and the whole family’s involvement in the treatment process immensely reduce the quality of life in the case of patients and their families. It also arises as a social problem by an increase in indirect costs necessary to fight the disease, such as doctor’s appointments, absence from work and school or hospitalizations [7,13,14]. The pathophysiology of atopic dermatitis is complex and multifactorial. Its understanding is complicated by the number of synergized factors that influence the disease. The most important are: genetic disorders, a defect in the epidermal barrier, an altered immune response and disturbed microbiological balance of the skin.

Additionally, the environmental aspects, such as increased exposure to airborne or food allergens, pollution, infections, use of antibiotics, breastfeeding duration, diet, cosmetics or strong detergents, should be considered [15,16]. Numerous complex changes at the genetic level, along with those at the level of innate and adaptive immunity, are the basis for characterizing the different phenotypes and endotypes ofatopic dermatitis and developing new diagnostic and therapeutic approaches. The following paper presents some of the molecular mechanisms involved in the pathogenesis of atopic dermatitis.

## 2. Genetic Defect and Epidermal Barrier

The initial suspicions of atopic dermatitis’s genetic background appeared along with the observations of a higher incidence of AD in families with atopy [17]. It has been noted that children of parents with a history of allergic diseases have a greater risk of developing AD [18,19,20]. In the case of parents who have asthma, allergic rhinitis or food allergies, the risk of developing AD in the offspring is 1.5 times higher. When one of the parents has atopic dermatitis, the risk of developing AD in their children increases 3-fold, while both parents increasing the risk 5-fold [18]. While examining the morbidity among monozygotic and dizygotic twins, AD’s risk was determined at 72–86% and 21–23%, respectively [21]. There are many cooperating genes responsible for the disease’s pathogenesis. However, this is not a simple Mendelian inheritance. Genes are also subject to various heredity phenomena such as epigenetic changes, incomplete gene penetrance, and genomic imprinting. Thirty-one different chromosomal loci containing AD susceptibility genes have been located in genome research [22]. Several groups have been distinguished: the most important are genes encoding structural and functional proteins of the epidermis and genes encoding proteins that regulate the innate and acquired immune response [23]. Gene mutations in the first group lead to impairment of the epidermal barrier function. The most popular of this group is the filaggrin gene mutation, considered one of AD’s major genes. The filaggrin gene is located in the epidermal differentiation gene complex (EDC) on the long arm of the 1q21 chromosome. The EDC contains 27 genes, 14 of which are expressed during the final keratinocyte differentiation process and are predominantly proteins in the cornified envelope. The remaining 13 genes located within the EDC are genes encoding proteins that are likely to play the role of signal transducers during keratinocytes’ differentiation processes and other cells and tissues [24]. The FLG (filaggrin) 2282del4 and R501X mutations are the Europeans’ major mutation variants. Both are null allele, which lead to the lack of production of protein encoded by the genes. Studies have confirmed that filaggrin mutations are a high-risk factor for AD and are related to an early-onset and severe phenotype [25]. It is worth emphasizing that the mutation may occur in asymptomatic patients and that the lack of the mutated gene does not protect against the disease. There are also discussions about the potential influence of FLG mutations on elevated IgE level, on provocation atopic march and asthma [25,26]. The gene’s starting product is profilaggrin—a highly phosphorylated histidine-rich molecule, which is the main component of keratohyalin granules. Filaggrin is formed from the insoluble and functionally inactive precursor molecule under the proteolytic activity of enzymes from the group of serine proteases (e.g., Caspase-14) [27]. The resulting FLG monomers aggregate keratin fibres through the catalytic activity of the transglutaminase-1 enzyme, which results in the flattening of cells. So-called corneocytes build the stratum corneum [28]. In addition to filaggrin, many other proteins build the cornified envelope, such as loricrin, involucrin, and small proline-rich proteins [24]. The corneocytes are the scaffold for the extracellular matrix of lipids. The entirety protects skin against excessive water loss, maintains the appropriate pH of the skin, inhibits the expansion of Staphylococcus aureus and limits the penetration of antigens to deeper layers. Further transformations and degradation of filaggrin lead to glutamine, histidine, alanine and their derivatives, such as pyrrolidone carboxylic acid (PCA) and urocanic acid (UCA) included in the natural moisturizing factor (NMF) [29]. Mutations leading to impaired protein synthesis cause increased transepidermal water loss (TEWL), excessive skin dryness, higher pH on the skin surface and disturbances in the proportions and amounts of free fatty acids, ceramides and triglycerides. The barrier defect causes degradation of intercellular connections, higher proteases activity, increased epidermal permeability, infiltration of antigens, and stimulation of proinflammatory cytokines. Genes encoding proteins of intercellular junctions (including claudins and occludins) belong to the group of genes responsible for the epidermal barrier’s integrity and proper function. Those transmembrane and intracellular proteins makeup complexes that connect adjacent cells called tight junctions [30,31]. They regulate the passage of ions, water and solutes.In the epidermis, they are mainly located in the granular layerand are responsible for differentiation and keratinization. Damage to those proteins leads to increased water loss, skin dryness, as well as to infiltration and presentation of antigens on Langerhans cells [32,33]. The AD patients presented decreased expression of tight junction proteins and inversed correlation of claudin-1 Th2 biomarkers [32]. The other genes involved in the pathogenesis of AD at the level of the epidermal barrier are genes encoding a serine protease inhibitor (SPINK-5 / LEKT1, cystatin A), genes encoding epidermal proteases: mast cell chymase gene (CMA1), epidermal chymotrypsin and trypsin gene, epidermal N-methyltransferase gene (responsible for the degradation of histamine) [23,34].

## 3. Epigenetic Changes

**Figure 1:**
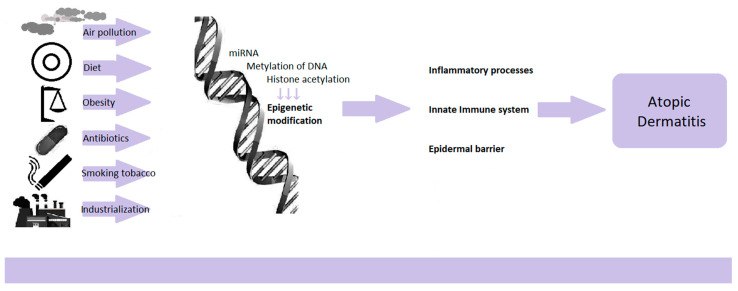
Epigenetic regulations dependent on environmental factors.

With the increase in allergic diseases in recent decades, the causes of this state of affairs have been sought. Genetic changes cannot explain such a rapid increase in the morbidity of atopic dermatitis. Changing environmental factors such as the Western lifestyle, industrialization, air pollution, diet change, obesity, increased use of antibiotics, and smoking are coming to the fore. The likely mechanism of how the environment affects the organism’s cells are epigenetic changes [35]. Epigenetics is the study of gene expression regulations that are not related to the modification of DNA sequences. The modifications lead to the activation or inhibition of the transcription of specific genes, resulting in the translation of the new mRNA into a polypeptide chain. Thus, they influence the functioning, activation and polarization of cells and the ability to secrete cytokines. Epigenetic modifications mainly consist of DNA methylation through miRNA and histone acetylation [23]. Importantly, changes in the epigenome may become permanent in the next generations, and the changing environment affects not only the postnatal but also the prenatal period (Figure 1).

A systematic review study of genetics and epigenetics in AD was conducted, with 11 papers on epigenetics. It has been confirmed with high probability that epigenetic regulation is one of the determinants of AD development, next to the FLG gene polymorphisms and genes related to the immune system and the skin barrier [36]. Research confirms epigenome differences between skin lesions in patients with AD and healthy people [23,35,37]. In AD, epigenetic changes concern genes known to affect the immune response regulation, genes of innate immunity, and genes encoding epidermis’s structural proteins (Figure 1).

DNA methylation is one of the most prevalent epigenetic mechanisms regulating gene expression [23]. The methylation process targets CpG (cytosine-phosphate-guanosine)-rich promoter sequences that indicate the direction and enable the transcription process. The addition of other methyl groups reduces the expression of the gene [23]. In an epigenome-wide association study in adult AD patients, significant differences in DNA methylation were observed at a total of 19 CpG sites and a correlation with altered gene transcript levels between epidermal lesions in AD patients and healthy control epidermis. These genes are mainly involved in keratinocyte differentiation, proliferation, and the innate immune response, including the S100A genes [37,38]. Activation of the GATA3 transcription factor in Th2 lymphocytes causes the production of IL-4, IL-5 and IL-13 by demethylation of the promoters of IL-13 and IL-4 genes as well as methylation of H3 histones in this region. It is accompanied by an increase in methylation of the IFG gene promoter and a decrease in acetylation H3 histones in this region of the gene [23]. Epigenetic changes in pregnant women have been studied extensively. For example, does exposure to smoke during the prenatal period induce methylation of umbilical cord blood DNA? Studies confirmed that high exposure to smoke might lead to hypomethylation of the TSLP 5’CpG island, positively correlated with AD [37]. Other prenatal environmental factors such as maternal allergy, maternal cytokine production and exposure to tobacco smoke can modify DNA methylation of the FOXP3 locus in umbilical cord blood. It causes babies with low Treg counts at birth and thereby favours exposure to the development of AD or allergy to food allergens in the first years of life [36].

Besides the transcriptional regulation of gene expression by chromatin modification, there is another mechanism—miRNA-mediated post-transcriptional regulation. MicroRNA (miRNA) is a class of small, evolutionarily conserved, non-coding molecules, single-stranded RNA. Specific sequences enable them to bind to specific mRNAs, resulting in mRNA degradation or translation inhibition [23,35,36]. The effects of action translate into the regulation of apoptosis, morphogenesis, proliferation, regulation of cellular metabolism, signal transduction, and cell differentiation [23,39]. In AD, they are involved in regulating the expression of genes determining Th2 polarization, the function of regulatory T lymphocytes, inflammatory processes, tight junctions, proliferation and apoptosis of epidermal keratinocytes, and synthesis of cytokines and chemokines [23]. Sonkoly et al. not only compared healthy skin and AD lesion but also identified 44 miRNAs that differed significantly between AD patients and healthy controls with 34 downregulated and 10 upregulated miRNAs. The authors also confirmed that miR-155 is significantly overexpressed in infiltrating T lymphocytes in AD skin lesions [40]. Infiltrating skin cells were found to express miR-155, and CD4+ T cells were the major cell type responsible for increased levels of miR-155 in skin lesions. Environmental factors such as mites and staphylococcal superantigens may induce miR-155 expression in atopic skin. MiR-155 can promote T cells’ activation and downregulate CTLA-4 expression, leading to chronic inflammation maintenance [37,41]. Another study showed that miR-223 level was elevated in whole blood cells in AD patients and that histamine-N-methyltransferase (HNMT), the major histamine-degrading enzyme, was increased in AD patients and murine AD models [42].

Summing up, it could be said that epigenetic regulation is a link between the changing environment and genetic changes, which jointly affect other pathogenic pathways, such as dysregulation of the immune system and disorders of the epidermal barrier.

## 4. Immunological Factors

**Figure 2:**
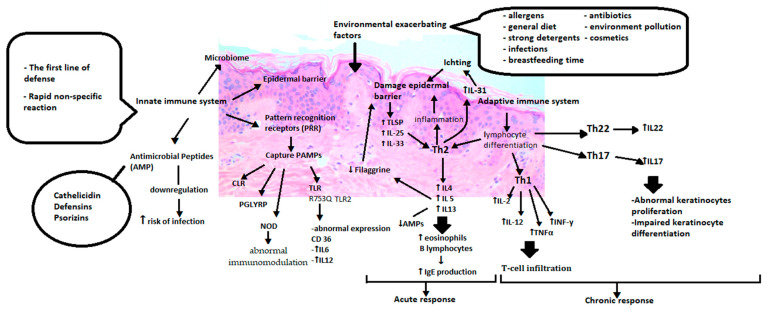
Immunological aberrations of atopic dermatitis. PRR (Pattern recognition receptors), PAMP (Pathogen-associated molecular patterns), CLR (C lectin receptors), PGLYRP (peptidoglycan recognition proteins), NOD (Nucleotide-binding oligomerization domain), TLR (Toll-like receptors), IL6 (Interleukin 6), IL12 (Interleukin 12),TSLP(Thymic stromal lymphopoietin), IL25 (Interleukin 25), IL33 (Interleukin 33), Th2 (T helper cells 2), IL4 (Interleukin 4), IL5 (Interleukin 5), IL13 (Interleukin 13), IL31 (Interleukin 31), Th1 (T helper cells 1), IL2(Interleukin 2), TNFα (Tumor necrosis factor alpha), INFγ (Interferon gamma), Th22 (T helper cells 22), IL22 (Interleukin 22), Th17 (T helper cells 17), IL17 (Interleukin 17).

While describing atopic dermatitis causes, it is impossible to avoid disorders of immune regulation. Consequently, two different hypotheses have been proposed, from inside to outside and from outside to inside. The first one suggests that immunological aberrations are believed to be the primary initial event in development, and stimulation with allergens leads to weakening the epidermal barrier. The latter hypothesis assumes that an impaired skin barrier is the first step in atopic eczema’s pathogenesis and is required for immune dysregulation to occur [43]. Figure 2 shows that the abnormalities start with the innate immune system, which is the body’s first line of defence and is responsible for rapid and non-specific protection against external factors that may turn out to be pathogenic (Figure 2).

It consists of the epidermal barrier, cells of the immune system, cytokines, pattern recognition receptors (PRR), antimicrobial peptides and skin microbes [44]. PRRs are responsible for distinguishing pathogen-associated molecular patterns (PAMP). These include toll-like receptors (TLR), proteins containing a nucleotide-binding oligomerization domain (NOD-like receptors or NLRs), a retinoic acid-induced gene, C-type lectin receptors (CLRs), and peptidoglycan recognition proteins (PGRPs). It has been noted that TLR polymorphisms such as TLR1 (rs5743571 and rs5743604), TLR6 (rs5743794) and TLR10 (rs11466617) and genetic variants TLR2 mutations may increase the susceptibility to AD by increasing colonization with Staphylococcus aureus [45,46]. Also, the relationship between R753Q in the TLR2 gene and severe AD is described [47]. On the other hand, NOD1 and NOD2, which belong to the NLPR family, depending on different genetic variants or mutations, may result in incorrect immunomodulation in allergic diseases. Significant associations of the NOD1 SNP rs2907748, rs2907749 and rs2075822 with IgE levels could also be observed. The rs2736726 and rs2075817 polymorphisms showed weak associations with atopic eczema. Analyses revealed a significant association between the NOD1 haplotype G-A-C-A-C-C-G-C-G-T-G and IgE as well as haplotype A-G-T-A-C-C-G-T-A-C-G and atopic dermatitis [48,49]. The meta-analysis included a total of nine case-control studies. It showed that the heterogeneous “GA” genotype of the TLR2 rs5743708 and “AG” genotype of the TLR4 rs4986790 might be associated with increased susceptibility to atopic dermatitis in Caucasians [50]. Heterozygous TLR2 R753Q carriers with AD, in comparison to healthy ones, showed modified CD36 expression after stimulation and increased production of IL-6 and IL-12 by monocytes after TLR2 stimulation [51].

Relationships between TLR2 expression and high-affinity IgE receptor (FcεRI) levels were also observed [52]. The FcεRI is found on various immune cells, binds immunoglobulin E (IgE) and plays a major role in allergic diseases [53]. The expression patterns of FcεRI and TLR2 have been found to correlate with total IgE levels [52,53]. FcεRI-mediated signals can prolong the survival of monocytes, thus contributing to chronic allergy. Bacterial infections induce pro-inflammatory cytokines by activating TLR2, which aggravates allergies. They can also enhance the upregulation of FcεRI, which further enhances the ongoing allergic reaction [52]. This assumption is supported by the study on mast cell reactivity under IgE-mediated activation with TLR ligand. Prolonged mast cells exposure to TLR ligands modulates effector responses, inducing them to increase the release of several inflammatory mediators when combined with rec. IgE [54]. The combined effect of the TLR2 (TLR2) rs4696480 gene, the FcεRI (FCER1A) rs2252226, and rs2251746 α chain gene polymorphism on the severity of AD was assessed. Higher SCORAD was observed in the major TLR2 rs4696480 homozygotes and simultaneously carried the smaller FCER1A rs2252226 allele [55]. The stimulation of human basophils by the parallel activation of Toll-like receptors and FcεRI directs the response towards the Th2-gated response [56]. Not all studies confirm a positive correlation between TLR and FcεRI. In one of the studies, the FcεRI level was reduced at the protein and mRNA levels after stimulation with TLR1/2 or TLR2/6 [57]. The expression of FcεRI likely depends on the stage of maturation and the type of cells and tissue in which they are located, the total level of serum IgE, and the various cofactors.

The skin produces antimicrobial peptides (AMP) to destroy or inhibit microbes’ growth. They include over 20 peptides with antibacterial activity, including cathelicidin, defensins and psoriasins. Altered AMP expression and secretion may contribute to increased susceptibility to skin infections by viruses, bacteria and fungi in AD patients [58,59].

Innate lymphoid cells (ILCs) are a unique family of immune effector cells that functionally resemble T cells, but they lack clonal antigen receptors. ILCs stimulate the production of cytokines and affect immune and non-immune cells in the local tissue environment. Innate lymphoid cells type 2 (ILC2) are known for their ability to secrete proallergic cytokines, including IL-4, IL-5, IL-9 and IL-13. This fact indicates that ILC2 may be involved in various allergic diseases by initiating a Th2 response [60,61]. The exact mechanism of ILC2 activation in atopic dermatitis remains under discussion. Patients with atopic dermatitis presented ILC2 infiltration in damaged skin [60,62,63,64]. Low levels of ILC2 in mice models of AD-like skin inflammation alleviated the inflammation [63]. Increased expression of various receptors such as IL-25 receptor, IL-33 ST2 receptor, TSLP receptor and PGD2 CRTH2 receptor on ILC2 cells has been observed in the skin of patients with atopic dermatitis. This suggests that ILC2 responds to nonspecific cellular origin factors such as IL-33, IL-25 and thymic stromal lymphopoietin (TSLP) or eicosanoids [60,61,64]. Some studies suggest the influence of ILC2 in acute inflammation and explain the increase in the number of ILC2 in tissues due to the general increase in infiltrating immune cell populations [62].

One of the characteristic phenomena is the preference for the differentiation of CD4 lymphocytes towards the Th2 lineage. Excessive production of Th2 lymphocytes leads to increased production of the cytokines IL-4, IL-5 and IL-13. Cytokines stimulate IgE antibodies and eosinophils in peripheral blood and tissues [65]. Inflammation damages the epidermal barrier, which overlaps with the primary defects of the barrier [66]. Factors influencing the destruction of the epidermis, such as damage, infections or ongoing inflammation, stimulate keratinocytes to produce proinflammatory cytokines such as TSLP (Thymic stromal lymphopoietin), IL-25 and IL-33. They also activate the Th2-mediated immune response [67]. TSLP, through its receptor (TSLPR), activates immature dendritic cells, enhances the maturation of antigen-presenting cells (APCs). Moreover, TSLP promotes the activity and chemotaxis of eosinophilia and enhances the expression of IL-4, IL-5 and IL-13 [68]. IL-25 induces the expression of various chemokines, such as eotaxin, TARC (CCL17, thymus and activation-regulated chemokine) and MDC (macrophage-derived chemokine), which are necessary for the recruitment of eosinophilia and Th2 cells [69]. IL-33 activates NF-kB (nuclear factor kappa-light-chain-enhancer of activated B cells) and MAP (mitogen-activated protein kinases) through the receptor, whichstimulates the production of cytokines related to Th2 response, such as IL-4, IL-5, and IL-13 [64]. Continuous IL-4 and IL-13 stimulation causea reduction of filaggrin expression in the epidermis [70]. Acute inflammation hinders the synthesis of other proteins involved in keratinocyte differentiation, translating into impaired barrier reconstruction [71,72]. Th1 lymphocyte chemotaxis and increased production of IL-2, IL-12, TNFα, and INF cytokines occurduring the development of disease in the chronic phase. It is also important to notice that the additional activation pathway through the Th22 and Th17 cytokine, which are releasing IL-17, IL-19 and IL-22, and regulatory lymphocytes’ role as another mechanism of atopic eczema, is widely discussed [73,74]. The proinflammatory cytokines IL-36α, IL-36β and IL-36γ and antagonists of their receptors IL-36Ra and IL-38 are signalling proteins belonging to the IL-1 family. The change in the newly discovered representatives’ regulation was observed in atopic diseases. Recent studies show that they may be involved in AD’s pathogenesis [75].

Atopic dermatitis is such a complex and diverse disease that different immune responses occur in various patient groups. Therefore, these specific molecular mechanisms underlying the disease have been defined as disease endotypes which set and variable constellations give rise to a particular phenotype. In acute lesions, there is an increase in cytokines from the Th2 and Th22 axes, and to a lesser extent, Th17. With the development of the disease process, increase in Th1 response biomarkers’, the Th2 and Th22 responses intensify [76]. Apart from the typical Th2-dependent response in the pediatric population, a targeted increase in Th17 and Th22 cytokines and a low level of Th1 cytokines is visible [76,77]. There is an increasing number of discussions concerning different endotypes depending on ethnic backgrounds. The Th2 and Th22 lineage responses with lower Th1 and Th17 dominate in the European and American populations [78]. Data suggest that Asian AD patients exhibit unique immune dysregulation in comparison to European and American patients. Thus, in the Japanese population, an increased frequency of the Th17 axis (and related IL-17A, IL-19, IL-22 and S100A12) and suppression of the Th1 axis is indicated [76]. In the Chinese population, apart from Th2 activation and the associated activation of the chemokines IL-4, IL-13, IL-5, IL-10, IL-31, increased Th17/IL-23 (e.g., IL-17F/IL-19/IL-21/CCL20) and increased expression of Th22-induced markers are noticeable [76,78]. African Americans with AD have shown targeted responses to Th2 and Th22, with parallel attenuation of Th1/Th17 [76].

Increased IgE levels and specific IgE in plasma are observed in the case of AD patients. The higher expression of FcεRI is noticeable in the affected skin [79]. These disorders are associated with higher levels of eosinophils and a family history of atopic disease. External irritants and damage to the epidermal barrier lead to the stimulation of the Th2 response. Next, B lymphocytes produce IgE antibodies specific against their proteins. Immunoglobulins bind to FcεRI on skin dendritic cells, enhancing the immune response and the skin’s inflammatory reaction [79]. There are ongoing discussions about whether auto-reactivity translates into a clinically significant autoimmune process. Three arguments oppose this hypothesis: thelack of correlation between IgE levels and disease severity, the ambiguous impact of eliminating air-related and food allergens, and cases of AD patients with an adverse history of atopic disease.

## 5. Pruritus Pathophysiology

Itching skin is one of AD’s main symptoms, significantly influencing patients’ quality of life. Improperly controlled pruritus limits daily activity, reduces productivity and disturbs sleep. The base of itching stems from the organised interactions between keratinocytes, the immune system and non-histaminergic sensory nerves. Additionally, emotional stress, sleep and alcohol consumption may intensify pruritus in AD patients [80,81]. Pruritogens promote itching by interacting with substance-specific receptors. The itching is mediated by unmyelinated C fibres and thinly myelinated Aδ fibres derived from cell bodies in the dorsal root ganglion (DRG) [81,82,83,84]. The brain processes the itching signal and triggers motor activity—scratching. Common immune disorders involved in the pathogenesis of AD also contribute to atopic pruritus. Th2 cells, eosinophils, neutrophils and mast cells release pro-inflammatory cytokines and peptides that activate pruritoceptive pathways [80,81,85,86]. One of the best-known mediators of pruritus is IL-31, which is produced by Th2 cells. Studies have shown increased levels of IL-31 in damaged skin of AD patients. Moreover, the pruritic effect of IL-31 has been confirmed on animal models [80,82,83]. IL-31 binds to IL-31A receptor (IL-31RA) on sensory neurons and causes itching by activating ion channels of TRPV1 (transient receptor potential vanilloid 1) and TRPA1 (transient receptor potential ankyrin 1). IL-31 also stimulates the lengthening and branching of nerve fibres, which may exacerbate the itching sensation [83]. Other cytokines cause pruritus and are involved in the pathogenesis of AD, namely IL-4 and IL-13. This is confirmed by the presence of IL-4 α and IL-13 α1 receptors on mice and human sensory neurons [81]. An additional role of IL-4 is a potential increase of pruritus by sensitizing neurons to other stimuli. Keratinocytes release certain pruritic factors. One of them is alarmin TSLP, which also directly stimulates neurons. The main role of TSLP is to activate the Th2-dependent pathway, stimulate the immune system, and produce pro-inflammatory cytokines. That proves that TSLP contributes indirectly to itching. Moreover, scratching of skin damages epithelial keratinocytes, which results in the release of inflammatory cytokines, direct and indirect activation of the Th2 axis and the release of pruritic cytokines from both keratinocytes and cells of the immune system. The re-binding of these itching factors to the sensory nerves triggers the urge to continue scratching. These feedback loops were named in AD “itch–scratch cycle” [80,84]. The role of histamine in the cause of itching in AD is unclear. Simultaneous blockade of H1R and H4R is more effective in reducing pruritus and inflammation than either of them alone. Non-sedative antihistamines used in clinical practice are not very effective in relieving pruritus in AD, which suggests the involvement of a non-histaminergic mechanism [81,85]. Apart from the mentioned factors, numerous other endogenous and exogenous pruritic factors are produced due to inflammation, such as interleukins, leukotrienes and endothelins [85,86]. It has been noticed that AD patients have an increased sensitivity to itching caused by stimuli unnoticeable for healthy people. This phenomenon is called neuronal sensitization [80,81,84,85,86], which is based on increasing the sensitivity to stimuli. In the case of changes in the properties of primary nerve endings, it is of peripheral sensitization. In the case of changes in the central nervous system, it is of central sensitization. Concerning AD, there is a change in the perception of itching. Allokinesis is an abnormal sensory state in which stimuli that do not usually itch as well as hyperkinesis are abnormal itching states in which a normally itchy stimulus lasts longer than usual [86]. Moreover, itching may persist even after the original provoking stimulus has been removed. The main cause of sensitization is the alteration of neurons at the peripheral level. The hypersensitivity of sensory neurons to itching stimuli is probably caused by local inflammation. In AD patients with skin lesions, the skin nerve fibres’ density and thickness were much greater than in the healthy control group. That would explain the significantly high severity of pruritus in patients with relatively mild eczema [85,86]. However, appropriate conventional treatment of AD should reduce the itching sensation. The conducted clinical trials confirm that the introduction of anti-inflammatory therapies, from topical to conventional inflammatory drugs and new biological drugs, improves the skin’s condition and helps to reduce itching [80,84,85,86].

## 6. Microbiome

The skin microbiota consists of all microorganisms on the surface of the epidermis. Usually, it is perceived as the bacterial flora inhabiting the skin. However, this conceptalso includes viruses, fungi, and protozoa [87]. The quantitative and qualitative composition of human microbiota is individual and varied due to its location on the human body. Numerous factors influence this variability:the different thickness of the skin, exposure to ultraviolet light, temperature, humidity, sebum content, different pH of the skin, the presence of natural cavities’ folds and protrusions of the body what divides the areas into high humidity, sebaceous and dry ones [88]. Microorganisms living on the skin create a mutual relations system and benefit the host. This phenomenon is known as commensalism. They participate in metabolic processes, maintain an appropriate immune status by influencing the innate and acquired immune system, protect against pathogenic microorganisms and support the epidermal barrier’s action [87,89]. Species diversity and high stability over time characterise the microbiome of healthy skin. The disturbance and dysfunction of various microorganisms lead to diseases, including atopic dermatitis [90,91]. It has been proven that the composition and diversity of microorganisms on the skin differ between people with eczema and those healthy ones. In atopic skin, there has been a reduction in commensal bacteria of the genera Streptococcus, Corynebacterium, Cutibacterium and the type Proteobacteria with the increase towards the genus Staphylococcus in general (*S. aureus* in particular) [92]. Changes in quantitative and qualitative microbiota may occur before the disease’s clinical manifestation [93]. One study suggested that normal microbiota staphylococcal commensals may modulate skin resistance and protect against AD’s development [94]. What is more, therapeutic interventions such as topical treatments with corticosteroids, calcineurin inhibitors, or even moisturizers and emollients in patients with atopic eczema may restore barrier function and normalize the skin microbiome [95,96]. Reduction of the microbiome’s diversity correlates with the severity of the disease and increased colonization by pathogenic bacteria.

**Figure 3:**
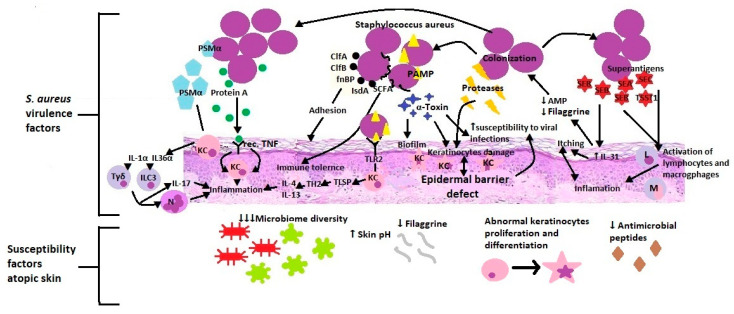
*S. aureus* virulence factors & atopic skin susceptibility factors. PSMα (Phenol-soluble modulins alpha), KC (Keratinocytes), IL-1α (Interleukin 1α), IL-36α (Interleukin 36α), Tγδ (Gamma delta T cells), ILC3 (Type 3 innate lymphoid cells), IL-17 (Interleukin 17), N (Neutrophils), Rec. TNF (receptors for Tumor necrosis factor), ClfA (Clumping factors A), ClfB (clumping factors B), fnBP (Fibronectin-binding protein), IsdA (Iron-regulated surface determinant A), SCFA, PAMP (Pathogen-associated molecular patterns), TLR2 (Toll-like receptor 2), Th2(T helper cells 2), IL-4 (Interleukin 4), IL-13 (Interleukin 13), AMP (Antimicrobial peptides), IL-31 (Interleukin 31), SEA (Staphylococcal enterotoxin A), SEB (Staphylococcal enterotoxin B), SEC (Staphylococcal enterotoxin C), TSST1 (toxic shock syndrome toxin 1), L (Lymphocytes), M (Macrophages).

Staphylococcus aureus is the main subject of microbiome research in AD development. It is a Gram-positive bacterium that inhabits the upper respiratory tract and skin. *S. aureus* appears on the skin of people with eczema, with a greater prevalence of lesions than healthy skin. It is also the most common pathogen causing skin infections’ clinical symptoms [97]. A higher colonization index and increased pathogen density show a positive correlation with the skin lesions’ severity and the severity of the disease [98,99]. This condition is associated with *S. aureus* accommodation to the inflamed substrate’s conditions. Colonization by pathogenic staphylococci is favoured by a reduced amount of filaggrin and corneocytes’ disturbed structure, higher than the standard skin pH and decreased production of antimicrobial peptides [92] (Figure 3).

Besides, adhesion factors located on the surface of the cell wall such as clumping factors A and B (ClfA, ClfB), fibronectin-binding protein (fnBP), and iron-regulated surface determinant A (IsdA) facilitate adherence to the stratum corneum. *S. aureus* integrates short-chain, unbranched fatty acids into its membrane, resulting in increased tolerance on *S. aureus*’ antigens of the body’s innate immunity [92]. Through the virulence mechanisms, *S. aureus* damages and penetrates the epidermal barrier, which intensifies inflammation, changes the immune response and promotes bacterial and viral infections. For example, staphylococcal alpha-toxin has various functions: forming a biofilm on the surface, hindering the elimination of bacteria, connecting with corneocytes, damaging one of the skin barrier components, and promoting the development of viral infections [92,97]. Additionally, *S. aureus* releases at least ten other proteases involved in the stratum corneum’s dissolution. Staphylococcus directly stimulates endogenous keratinocyte serine proteases and metalloproteinases in skin fibroblasts [99,100]. All the resulting products weaken, destroy and increase the epidermal barrier’s permeability. Upon dissolution, cell wall-bound protein A triggers keratinocytes’ inflammatory response through the TNF receptor. *S. aureus* secretes staphylococcal enterotoxin A, B and C as well as toxic shock syndrome toxin 1 (TSST-1), which act as superantigens and trigger the uncontrolled activation of lymphocytes and macrophages [99]. What is more, Staphylococcal enterotoxin B increases the expression of IL-31, which causes itching in AD. IL-31 also inhibits filaggrin and AMP expression, resulting in increased colonization of *S. aureus*. Additionally, IL-31 serves as a critical immune-neuron link between Th2 cells and sensory nerves in T cell-mediated pruritus development. Itching and scratching are important factors in initiating and sustaining inflammation in a further step [83]. *S. aureus* recognized by the use of PAMP (proinflammatory staphylococcal lipoproteins) stimulates (through TLR2) the epidermis to produce TSLP and a Th2-dependent inflammatory response [97,98]. The produced bacterial virulence factor (PSMα-Phenol-soluble modulins) activates keratinocytes to produce IL-1α and IL-36α, which in turn stimulates Tγδ and ILC3 lymphocytes to release IL-17 and recruit neutrophils [101,102,103]. These studies suggest that mediators produced by *S. aureus* facilitate adherence, colonization, and propagation to the skin. Patients deficient in filaggrin may be particularly prone to expanding *S. aureus* due to an epidermal barrier defect and further consequences.

## 7. Summary

**Figure 4:**
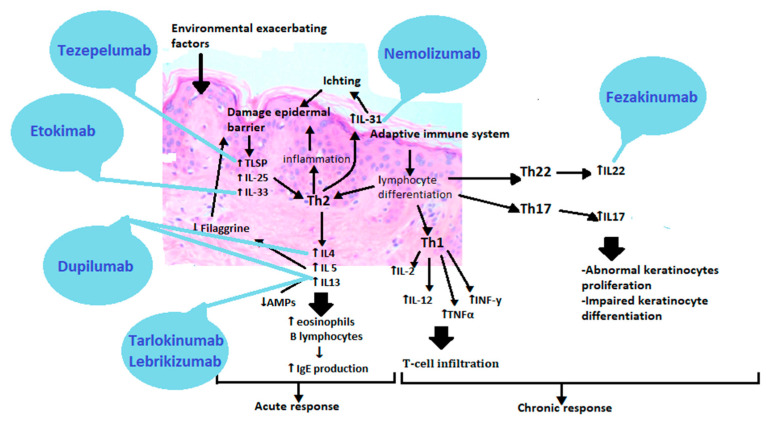
Therapies based on the pathogenesis of atopic dermatitis. TSLP(Thymic stromal lymphopoietin), IL25 (Interleukin 25), IL33 (Interleukin 33), Th2 (T helper cells 2), IL4 (Interleukin 4), IL5 (Interleukin 5), IL13 (Interleukin 13), IL31 (Interleukin 31), Th1 (T helper cells 1), IL2(Interleukin 2), TNFα (Tumor necrosis factor alpha), INFγ (Interferon gamma), Th22 (T helper cells 22), IL22 (Interleukin 22), Th17 (T helper cells 17), IL17 (Interleukin 17).

Atopic dermatitis is a complex disease with a heterogeneous clinical picture, in which a myriad of overlapping factors is involved in the pathogenesis. These are the mutations of the epidermal genes, skin barrier dysfunction, immunity disorders, changes in the lipid composition and microbial imbalance. Thanks to current advances and science, understanding the molecular basis of atopic dermatitis allows for its breakdown into different phenotypes. Emerging treatments are based on the action of specific molecules involved in eczema’s pathophysiology [104]. The development of targeted, endotype-specific (barrier-types, immunotypes, genotypes) therapies could open up a new, promising era for personalized treatment of atopic dermatitis [105]. However, the targeted medicines in atopic dermatitisare still at a very early stage compared to other diseases (Figure 4).

The first approved biological preparation is a human antibody that binds to the alpha subunit of IL-4 and IL-13 receptors. It is called Dupilumab [105,106]. More drugs are on the horizon, bringing us closer to patient-centred medicine, such as anti-IL-13 antibodies—Tralokinumab and Lebrikizumab, anti-IL-31 receptor antibody—Nemolizumab, anti-IL-22 antibody—Fezakinumab, IgG1 anti-IL monoclonal antibody—33-Etokimab or anti-thymic stromal lymphopoietin antibody (anti-TSLP)—Tezepelumab [107,108]. Such a vast repertoire of drugs would allow selecting an appropriate preparation for patients depending on the presence or absence of a filaggrin mutation or the dominant immunotypes of Th2, Th17 or Th22. Janus kinase inhibitors act less selectively than orally and topically administered cytokine-targeting antibodies, but they are of great interest. By inhibiting the JAK-STAT pathway, they inhibit the transmission of information to the cell nucleus, which results in the inhibition of the production of many proinflammatory cytokines. These include Abrocitinib, Baricitinib, Upadacitinib, Tofacitinib, Ruxolitinib, Delgocitinib [109]. New therapies focus on improving the skin barrier function and restoring the immune balance found in AD. Understanding the disease is a continuous path into the unknown, but thanks to existing reports and research into new drugs, we are on the threshold of a new era of treatment.
